# CircCDYL Acts as a Tumor Suppressor in Wilms’ Tumor by Targeting miR-145-5p

**DOI:** 10.3389/fcell.2021.668947

**Published:** 2021-08-17

**Authors:** Rui Zhou, Wei Jia, Xiaofeng Gao, Fuming Deng, Kai Fu, Tianxin Zhao, Zhongmin Li, Wen Fu, Guochang Liu

**Affiliations:** Department of Pediatric Urology, Guangzhou Women and Children’s Medical Center, Guangzhou Medical University, Guangzhou, China

**Keywords:** microRNA, tight junction protein 1, Wilms’ tumor, circular RNA, mir-145-5p, biomarker

## Abstract

Circular RNAs (circRNA) have been reported to exert evident functions in many human carcinomas. However, the possible mechanisms concerning the circRNA in various tumors are still elusive. In this research, we analyzed the expression profile and biological functions of circular RNA CDYL (circCDYL, circBase ID: hsa_circ_0008285) in Wilms’ tumor. Here, miRNA and gene expression were examined by real-time PCR in Wilms’ tumor tissues and cell lines. The functions of circCDYL and its potential targets to influence cell proliferation, migration, and invasion in Wilms’ tumor cells were determined by biological functional experiments *in vitro* and *in vivo*. We predicted and analyzed potential miRNA targets through online bioinformatic tools. To validate the interactions between circCDYL and its targets, we performed RNA fluorescence *in situ* hybridization, biotin-coupled miRNA capture assay, and biotin-coupled probe pull-down assay. Tight junction protein l (TJP1) was proved to be the target gene of the predicted miRNA by dual-luciferase reporter assay. The expression level of TJP1 in Wilms’ tumor cells was identified *via* Western blot. We showed that circCDYL was downregulated in WT tissue compared with adjacent non-tumor tissue. Upregulation of circCDYL could reduce cell proliferation, migration, and invasion. Mechanically, circCDYL, functioning as a miRNA sponge, decreased the expression level of miR-145-5p and TJP1 3′UTR was validated as the target of miR-145-5p, facilitating the circCDYL/miR-145-5p/TJP1 axis. In conclusion, our study suggested circCDYL as a novel biomarker and therapeutic target for WT treatment.

## Introduction

Wilms’ tumor (WT, nephroblastoma) accounts for about 6% of childhood tumors and for 95% of pediatric kidney tumors, ranking the fifth in childhood malignancy. Most WT develop before the age of 3 years (Wang et al., 2012). The recrudescence rate of WT is up to 15%, and the long-term survival rate for patients with relapsed WT is only 50%. Although in recent years, with the improvement of comprehensive therapy combining surgery, chemotherapy, and radiotherapy, nearly 85% of WT children were cured, a few children still died because of recurrence, metastasis, resistance to chemotherapy, and other complicated factors. Due to continuous research of WT in recent years, people have gained a better understanding of the pathogenesis of WT, but there are still many issues that deserve further discussion.

Circular RNA (circRNA) is a novel subtype of non-coding RNA with covalently closed loop containing the joint point of its 3′ end and 5′ end (Panda et al., 2017). Although circRNAs was firstly discovered more than 40 years ago, they drew researchers’ attention recently. Over 30,000 circRNAs have been identified through the high-throughput sequencing method. Due to the loop structure, exonuclease could hardly destroy circRNA. But their parental linear RNAs are sensitive to exonuclease. Thus, circRNAs are found abundant in mammalian cells due to their stability. CircRNAs were reported to be disease-specific and stage-specific in various pathologic environments (Salzman et al., 2013), which indicates that circRNAs can be potential novel diagnostic and therapeutic biomarkers (Fu et al., 2018). CircRNAs have been identified to be closely relevant with tumorigenesis. Increasing numbers of circRNAs have been identified to play a crucial part in the epithelial mesenchymal transition, indicating that they may influence the proliferation, migration, and invasion of tumor (Conn et al., 2015). Serman et al. (2014) reported that circITCH is upregulated in lung carcinoma tissues and acts as a sponge of miR-214; it then downregulated the activation of the Wnt/β-catenin signaling pathway. Yuan et al. (2019) reported that Cdr1as functions as miR-1270 sponge, upregulates APAF1 expression, and decreases cisplatin resistance in bladder cancer.

In this study, through deep sequencing in WT tissues, we identified has_circ_0008285, also called circCDYL, which was downregulated in WT tissues. CircCDYL, a circular RNA derived from the fourth exon of CDYL gene, is mainly located in cytoplasm and had no intronic sequence, IREs and ORFA (Liang et al., 2020). Previous research showed that circCDYL inhibits the oncogene C-MYC, and it restrains cell proliferation and migration in bladder cancer (Sun et al., 2019). Herein, we revealed that circCDYL was downregulated in WT and that the overexpression of circCDYL suppresses proliferation, migration, and invasion in WT both *in vitro* and *in vivo*.

## Materials and Methods

### Cell Culture

The WT cell line SK-NEP-1 and G401 was obtained from American Type Culture Collection (Manassas, VA, United States) and was cultured using McCoy’s 5A modified medium (30-2007) containing 15% FBS.

### Clinical Specimens

A total of 25 paired WT tissues and matched adjacent tissues were obtained from patients who were diagnosed as WT by histopathological examination in the Guangzhou Women and Children’s Medical Center, Guangzhou Medical University, between April 2017 and May 2019. Samples obtained were classified in accordance with the WHO criteria for WT. All the patients received neoadjuvant chemotherapy with a VA plan (Vincristine 1.5 mg m^–2^, once a week, for 4 weeks; Actinomycin D 15 g kg^–1^ D^–1^, once daily, for the first 5 days) performed before surgery. No patients received radiotherapy or targeted therapy before the operation. Guangzhou Women and Children’s Medical Center Ethics Committee approved this study (grant no. 43600). All patients’ legal guardians provided written informed consent prior to enrollment in the study. Resected tumors were frozen in liquid nitrogen immediately and stored at −80°C condition.

### RNA Extraction, Reverse Transcription (RT), Sanger Sequencing, and RT-qPCR

The nuclear and cytoplasmic RNAs were isolated according to the manufacturer’s instructions (Rio et al., 2010). Both qRT-PCR and microRNA (miRNA) RT-PCR methods referred to previous published paper (Yuan et al., 2019). The primer sequences were listed in Supplementary Table 1. All primers were purchased from RiboBio Co. Ltd. (Guangzhou, China). The relative RNA abundance was determined using the 2^–Δ^^Δ^^Ct^ method.

### RNase R Digestion

RNase R (4 U per 2 mg RNA) was used to treat RNA samples for 20 min at 37°C (Epicentre Biotechnologies, United States). RNA abundance was tested by RT- qPCR.

### Transfection

CircCDYL-overexpressing adenovirus was designed by Hanbio (Shanghai, China). MiR-145-5p mimics/inhibitors and small-interfering (si) tight junction protein 1 (TJP1) were designed and synthesized by GenePharma (Suzhou, China). According to the manufacturer’s instructions, Lipofectamine 3000 (Invitrogen, United States) was used during transfection.

### CCK-8 Assay and Cloning Formation Assay

After cells were supplemented using Cell Counting Kit-8 (CCK-8) (Dojindo, Kumamoto, Japan) for 120 min, wavelength of 450 nm was selected to detect the absorbency of cells.

In colony formation assay, the procedures were utilized in accordance with the publication (Li et al., 2018).

### Ethynyl-2-Deoxyuridine (EdU) Assay

Briefly, 100 μl of 50 μM EdU (RiboBio Co. Ltd., Guangzhou, China) was added into individual wells containing transfected cells, and they were incubated at 37°C for 2 h. Fluorescence microscopy was utilized to detect the proliferation.

### Wound Healing Assay

After infected cells grew to 100% confluence in six-well plates, the monolayer was scratched by a sterile pipette tip. Then, PBS was utilized to wash the wells for three times and serum-free medium was added into the wells. At 0 and 24 h, the width of the wound was photographed using DM2500 bright field microscope (LEICA, Wetzlar, Germany). The ImageJ software was utilized to quantify the cell migration capacity.

### Transwell Assays

The lower chambers were precoated with 100 μl of Matrigel (BD Bioscience, San Jose, CA, United States) for 30 min before the addition of medium to the chambers. Transfected cells (2 × 10^5^) were placed with serum-free McCoy’s 5A modified medium to the upper wells (8 μm membrane). Full culture medium (500 μl) was added into the bottom well. After incubation at 37°C for 24 h, cells in the upper surface were removed. Methanol was utilized to fix the cells stuck to the lower surface. Crystal violet (0.1%) was used to stain the cells. Then, cells were photographed using a microscope at 100 × magnification (Olympus, Japan).

### Flow Cytometry Analysis for Cell Cycle

The transfected cells were stained by Cell Cycle and Apoptosis Analysis Kit (Beyotime Biotechnology, Shanghai, China) and detected by flow cytometry. After that, the cell cycle was analyzed using a FACS Calibur flow cytometer (BD Biosciences, San Jose, CA, United States).

### Xenografts in Mice

The animal studies were approved by the Animal Management Committee of Guangzhou Medical University. G401 cells were transfected with the circCDYL adenovirus and GFP vector. Athymic BALB/c nude mice (4 weeks old, *n* = 8) were used. The mice were subcutaneously injected with 10^7^ G401 cells infected with circCDYL overexpressing adenovirus or control. Tumor volumes were tested 10, 15, 20, 25, and 30 days after injection.

### Prediction of miRNA Binding Sites of circCDYL

Databases, including miRanda,^1^ MicroTar,^2^ and RNAhybrid,^3^ were utilized to predict miRNAs that may be the downstream regulatory molecule of circCDYL.

### Dual-Luciferase Reporter Assay

The procedures were performed as before described (Yuan et al., 2019). GenePharma (Shanghai, China) synthesized the sequences corresponding to the 3′-UTR of TJP1 mRNA and containing the wild-type or mutated miR-145-5p binding sequence.

### RNA Binding Protein Immunoprecipitation Assay

The Magna RIP Kit (Millipore, Danvers, MA, United States) and Ago2 antibody (Cell Signaling Technology) were utilized to conduct the assay, and the procedures were performed as described before (Yuan et al., 2019).

### Biotin-Coupled Probe Pull-Down Assay

GenePharma (Shanghai, China) designed and synthesized the specific biotinylated probes to bind circCDYL’s junction area, and an oligo probe was synthesized as control. The sequences were as follows: circCDYL: 5′-CAATCCTTTCAACCTTTCCCGTTAAC-3′-Biotin, miR-145-5p: 5′- AGGGATTCCTGGGAAAACTGGAC-3′-Biotin, and Oligo: 5′-TGTCTGCAATATCCAGGGTTTCCGATGGCACC-3′-Biotin. The procedures were performed as described before (Yuan et al., 2019).

### Fluorescence *in situ* Hybridization (FISH)

GenePharma (Shanghai, China) designed and synthesized the DNA oligo probes for circCDYL (5′-CAATCCTTTCAACCTTTCCCGTTAAC-3′-Cy5) and for miR-145-5p (5′-AGGGATTCCTGGGAAAACTGGAC-3′-FAM). All procedures were performed as described before (Yuan et al., 2019).

### Immunohistochemistry

TJP1 antibody (1:500, ab221547, Abcam) was used to measure the expression of TJP1 in WT tissues. The procedures were performed as described previously (Yuan et al., 2019).

### Western Blot

The procedures were performed as described previously (Yuan et al., 2019). The primary antibody information is listed below: TJP1 (ab221547, Abcam) and β-actin (ab6276, Abcam).

### Statistical Analysis

The data were presented as mean ± standard deviation. The experiments were performed thrice independently. Statistical significances were determined using chi-square test, Student’s *t*-test, and two-way ANOVA. SPSS and GraphPad Prism analyzed the data, and *p* value < 0.05 was set as significance threshold.

## Results

### CircCDYL Is Downregulated in WT Tissue

**FIGURE 1:**
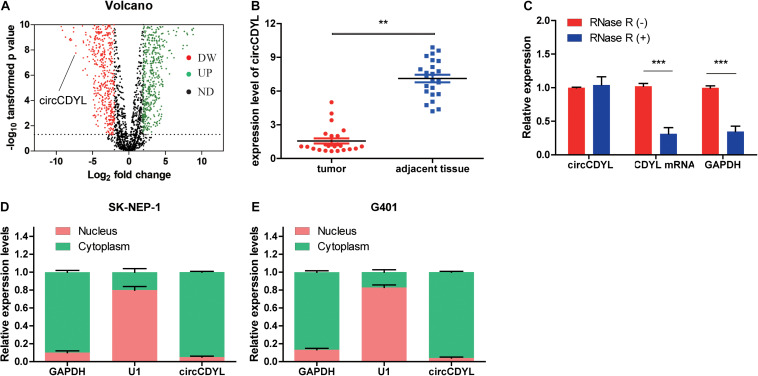
CircCDYL is downregulated in WT tissue. **(A)** Volcano plot of differential circular RNA expression in WT tissue. The abscissa indicated logFC, and the ordinate indicated the differential log10 *P*-value. The gene was represented as a dot. The green dot indicated the upregulated gene, while the red dot indicated the downregulated gene. **(B)** The relative expression of circCDYL in WT tissue and adjacent normal tissue. **(C)** The relative expressions of circCDYL, mRNA CDYL, and GAPDH after treatment with RNase R. **(D,E)** circCDYL was mainly localized in the cytoplasm in both SK-NEP-1 and G401 cell lines. GAPDH and U1 were used as negative control, respectively. Student’s *t*-test with two biologically dependent or independent replicates was used to determine statistical significance; ***P < 0.001, **P < 0.01, *P < 0.05.

The circCDYL expression was significantly downregulated in five WT samples compared with adjacent non-tumor tissues from the results of circRNAs deep sequencing (Figure 1A). RT-qPCR was utilized to determine the circCDYL expression in 25 WT tissues and their adjacent normal tissues from patients after radical nephrectomy. The results showed that circCDYL, but not CDYL mRNA (Supplementary Figure 1A), was significantly downregulated in WT tissues (Figure 1B). CDYL mRNA was found to be downregulated in SK-NEP-1 cells after RNA R treatment, but circCDYL was not downregulated (Figure 1C). By using the separation of nuclear and cytoplasmic RNA isolation, we demonstrated that circCDYL predominately localized in the cytoplasm in both SK-NEP-1 and G401 cell lines (Figures 1D,E). Data in Table 1 indicated that the increased circCDYL expression in WT had correlation with smaller tumor size and lower clinicopathologic stage, suggesting that circCDYL is associated with WT growth and metastasis.

**TABLE 1:** Correlation between circCDYL expression and clinicopathological characteristics of WT patients.

Clinicopathological characteristics	Cases (25)	circCDYL expression		*P*
		High (*n* = 10)	Low (*n* = 15)	
**Gender**				0.806
Male	13	6	7	
Female	12	4	8	
**Age, years**				0.934
<3	14	5	9	
≥3	11	5	6	
**Tumor size, cm**				0.037*
<8	10	7	3	
≥8	15	3	12	
**Histological type**				0.934
Favorable	11	4	7	
Unfavorable	14	6	8	
**NWTS-5 Clinicopathologic staging**				0.044*
I	8	6	2	
II–V	17	4	13	

### Overexpression of circCDYL Suppresses Proliferation, Migration, and Invasion in WT Cells

**FIGURE 2:**
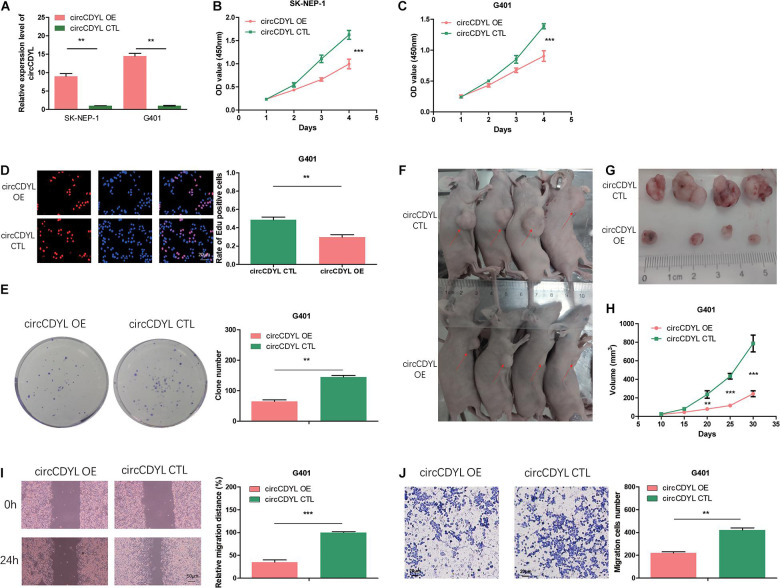
Overexpression of circCDYL suppresses proliferation, migration, and invasion in WT cells. **(A)** The expression levels of circCDYL were assessed using qRT-PCR in SK-NEP-1 and G401 cells after transfection with adenovirus carrying circCDYL. **(B,C)** The proliferation of SK-NEP-1 and G401 cells by the CCK-8 method. **(D,E)** The proliferation of G401 cells by EdU and colony formation assays. **(F,G)** Xenograft tumors in a mouse model of Wilms’ tumor. **(H)** Tumor volumes of the circCDYL overexpression and control treatment groups. **(I,J)** The migration and invasion of G401 cells by wound healing assays and transwell assays. Student’s *t*-test with two biologically dependent or independent replicates was used to determine statistical significance; ***P < 0.001, **P < 0.01, *P < 0.05.

To reveal the role of circCDYL in WT, we transfected the WT cell lines SK-NEP-1 and G401 with the adenovirus carrying circCDYL to increase circCDYL expression (Figure 2A). CDYL mRNA expression level was not influenced (Supplementary Figure 1B). Overexpressed circCDYL decreased the cell proliferation ability by CCK-8 assay (Figures 2B,C). As shown in Figure 2D, the rates of EdU positive cells were higher in control groups. CircCDYL significantly repressed the cell proliferation through EdU assay. Representative photographs of colony assay similarly showed that the upregulated circCDYL restrained cell growth compared to control group (Figure 2E). Furthermore, the *in vivo* assay was carried out to explore the function of circCDYL in tumor formation. The result showed that downregulation of circCDYL reduced tumor formation in G401 cells (Figures 2F–H). Flow cytometry showed that circCDYL overexpression could lead to S phase reduction (Supplementary Figures 2A–C). In addition, wound healing assays and transwell assays were performed to examine cell migration and invasion. CircCDYL overexpressing cells showed decreased ability in cell migration and invasion (Figures 2I,J).

### CircCDYL Acts as a Molecular Sponge for miR-145-5p

**FIGURE 3:**
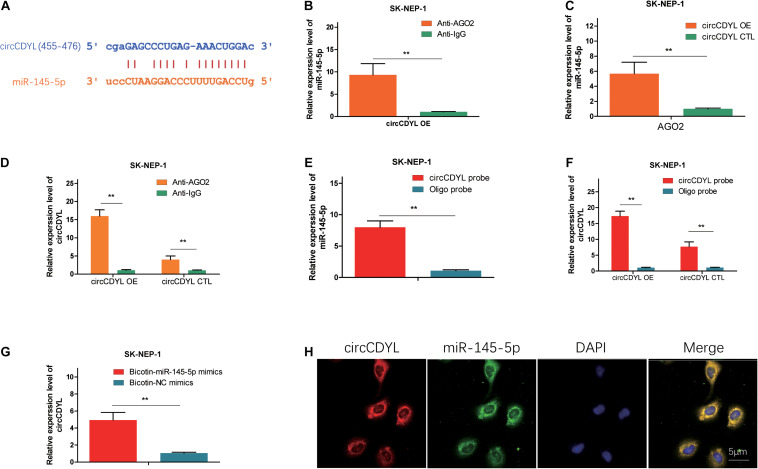
CircCDYL acts as a molecular sponge for miR-145-5p. **(A)** Possible binding sites between circCDYL and miR-145-5p. **(B)** The amount of circCDYL in circCDYL OE or circCDYL CTL groups was detected by an Ago2 RIP assay. **(C)** The relative enrichment level of miR-145-5p immunoprecipitated by Ago2 in circCDYL OE or CTL groups. **(D)** The relative enrichment level of miR-145-5p in the circCDYL OE group immunoprecipitated by Ago2 or IgG. **(E)** The relative enrichment level of miR-145-5p pulled down by biotinylated circCDYL probes or Oligo probes in the circCDYL OE group. **(F)** The relative enrichment level of circCDYL pulled down by biotinylated circCDYL probes or Oligo probes in the circCDYL OE or circCDYL CTL groups, respectively. **(G)** The relative enrichment level of circCDYL pulled down by biotinylated miR-145-5p mimics or non-sense control (NC) mimics in the circCDYL OE group. **(H)** circCDYL and miR-145-5p in G401 cells were colocalized by FISH. circCDYL was stained red, miR-145-5p was stained green, and the nuclei were stained blue (DAPI). Data represent the mean ± SD from three independent experiments. Student’s *t*-test with two biologically dependent or independent replicates was used to determine statistical significance; ***P < 0.001, **P < 0.01, *P < 0.05.

We found that miR-145-5p could bind to circCDYL, and the sites are shown in Figure 3A. AGO2 RNA immunoprecipitation (RIP) assay showed that AGO2 adsorbed more circCDYL than IgG (ninefold) in circCDYL overexpression cells (Figure 3B). The miR-145-5p levels pulled down by Ago2 in circCDYL OE cells were fivefold more than that in circCDYL CTL-transfected cells (Figure 3C). In circCDYL OE cells, more miR-145-5p was observed in the Ago2 pulldown pellet than in the IG pull-down products (Figure 3D). The biotin-coupled probe pull-down assay was later conducted to confirm the interaction furtherly. Compared with the Oligo group, specific enrichment of circCDYL and miR-145-5p was detected in the circCDYL pulled down eluents (Figures 3E,F). The phenomenon indicated that circCDYL could directly sponge miR-145-5p. To confirm the sponge functions of circCDYL, we performed biotin-coupled miRNA capture and fluorescence *in situ* hybridization (FISH). Biotin-coupled miR-145-5p analogously captured more circCDYL than biotin-coupled NC. This result indicated that miR-145-5p could attach itself to circCDYL (Figure 3G). CircCDYL (red) and miR-145-5p (green) could be photographed as colocalization in G401 cells by FISH assay (Figure 3H). The evidence above suggest that circCDYL could function as a sponge for miR-145-5p in WT.

### MiR-145-5p Enhances Proliferation, Migration, and Invasion in WT Cells

**FIGURE 4:**
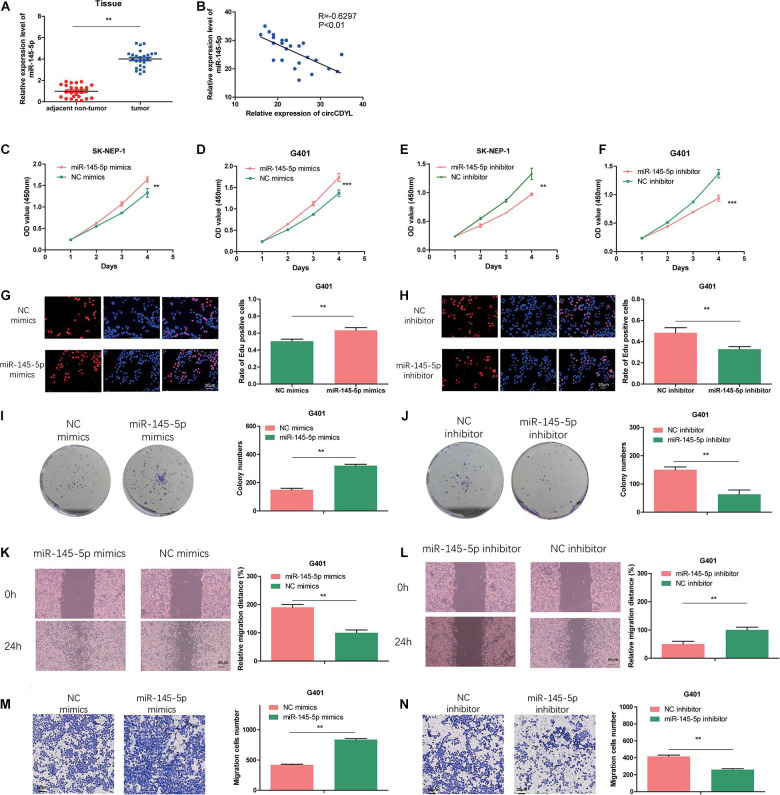
MiR-145-5p enhances proliferation, migration, and invasion in WT cells. **(A)** The relative expression of miR-145-5p in tumor and adjacent non-tumor tissue by RT-qPCR. **(B)** The relative expression of miR-145-5p had a negative correlation with circCDYL in the WT tissue. **(C–F)** The proliferation of SK-NEP-1 and G401 cells transfected by miR-145-5p mimics, miR-145-5p inhibitor, and their controls by the CCK-8 method. **(G–J)** The proliferation of G401 cells transfecting miR-145-5p mimics, miR-145-5p inhibitor, and their controls by EdU and colony formation assays. **(K–N)** The migration and invasion ability of G401 cells transfecting miR-145-5p mimics, miR-145-5p inhibitor, and their controls by wound healing assays and transwell assays. Data represent the mean ± SD from three independent experiments. Student’s *t*-test with two biologically dependent or independent replicates was used to determine statistical significance; ***P < 0.001, **P < 0.01, *P < 0.05.

We examined the miR-145-5p expression in 25 pairs of the tumor tissues *via* qRT-PCR. As illustrated in Figure 4A, the abundance of miR-145-5p in WT tissues was significantly upregulated. Correlation analysis showed that circCDYL expression level was of a negative correlation with miR-145-5p (*R* = −0.6297, *P* < 0.01, Figure 4B). MiR-145-5p overexpression in WT cell lines increased the proliferation rate (Figures 4C,D,G,I) and migration and invasion ability (Figures 4K,M) compared with cells in the NC mimics group. MiR-145-5p inhibited cells showed decreased proliferation rate (Figures 4E,F,H,J) and migration and invasion ability (Figures 4L,N) compared with cells in the NC inhibitor group.

### TJP1 Is a Direct-Target Gene of miR-145-5p, Which Could Reduce Proliferation, Migration, and Invasion in WT Cells

**FIGURE 5:**
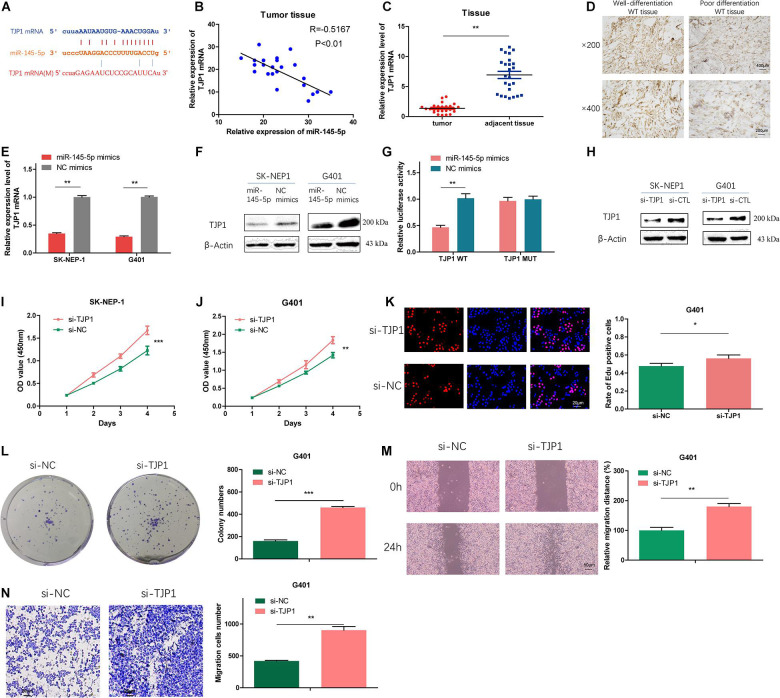
TJP1 is a direct-target gene of miR-145-5p, which could reduce proliferation, migration, and invasion in WT cells. **(A)** Possible binding sites between TJP1 mRNA and miR-145-5p. **(B)** The relative expression of TJP1 mRNA had a negative correlation with miR-145-5p in the WT tissue by RT-qPCR. **(C)** The relative expression of TJP1 mRNA in tumor and adjacent non-tumor tissue by RT-qPCR. **(D)** TJP1 expression in WT tissue by immunochemistry. **(E)** The expression of TJP1 mRNA determined by RT-qPCR in SK-NEP-1 and G401 cells overexpressing miR-145-5p. **(F)** TJP1 was the direct target of miR-145-5p through dual-luciferase reporter assays. **(G)** The expression of TJP1 protein by Western blot in SK-NEP-1 and G401 cells overexpressing miR-145-5p. **(H)** The expression of TJP1 protein by Western blot in SK-NEP-1 and G401 cells transfected by si-TJP1 or si-NC. **(I,J)** The proliferation of SK-NEP-1 and G401 transfected by si-TJP1 or si-NC cells by the CCK-8 method. **(K,L)** The proliferation of G401 cells transfected by si-TJP1 or si-NC by EdU and colony formation assays. **(M,N)** The migration and invasion of G401 cells transfected by si-TJP1 or si-NC by wound healing assays and transwell assays. Data represent the mean ± SD from three independent experiments. Student’s *t*-test with two biologically dependent or independent replicates was used to determine statistical significance; ***P < 0.001, **P < 0.01, *P < 0.05.

To further investigate the potential targets of miR-145-5p, we searched the online databases (starBase, miRWalk, miRDB, and TargetScan). TJP1 was one of the potential targets. The possible binding sites between TJP1 and miR-145-5p are shown in Figure 5A. Correlation analysis revealed that the expression of TJP1 mRNA showed a negative correlation with miR-145-5p (*R* = −0.7167, *P* < 0.01, Figure 5B). TJP1 mRNA level was downregulated in WT tissue compared with adjacent normal tissue by RT-qPCR (Figure 5C). Immunohistochemistry assay showed lower TJP1 protein expression in poor differentiation WT tissue (Figure 5D). TJP1 mRNA and protein were found to be downregulated in cells overexpressing miR-145-5p mimics on RT-qPCR and Western blot (Figures 5E,F). We performed the dual-luciferase reporter assay, and it revealed that co-transfection of miR-145-5p mimics and reporter plasmids significantly reduced the luciferase activity. On the contrary, we observed no obvious effect on the luciferase activity after co-transfecting mutated vectors and miR-145-5p mimics (Figures 5A,G). Consequently, the results proved that TJP1 is a direct target gene of miR-145-5p.

To investigate the effects of TJP1 on WT cells, we transfected si-TJP1 or si-CTL into SK-NEP-1 and G401 cells and the protein level of TJP1 was subsequently detected by Western blot. The results revealed reduced TJP1 expression (Figure 5H). Cells transfected with si-TJP1 showed increased cell proliferation rate (Figures 5I–L) and migration and invasion ability (Figures 5M,N), compared with cells in the si-CTL group. The same phenomena were observed in miR-145-5p overexpressing WT cells, which proved that miR-145-5p regulates cell proliferation, migration, and invasion in WT by targeting TJP1.

### MiR-145-5p Can Partially Reverse the Effect of circCDYL on Proliferation, Migration, and Invasion in WT Cells

**FIGURE 6:**
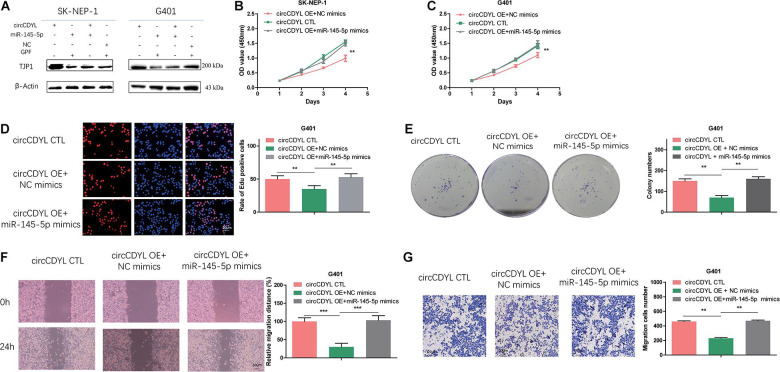
MiR-145-5p can partially reverse the effect of circCDYL on proliferation, migration, and invasion in WT cells. **(A)** Co-transfection of miR-145-5p mimics and circCDYL to detect the protein levels of TJP1 in WT cell lines by Western blot. **(B–G)** Co-transfection of miR-145-5p mimics and circCDYL to investigate malignant transformation of cells by CCK-8 **(B,C)**, EdU **(D)**, colony formation **(E)**, wound healing **(F)**, and transwell **(G)** assays in the WT cell lines. Data represent the mean ± SD from three independent experiments. Student’s *t*-test with two biologically dependent or independent replicates was used to determine statistical significance; ***P < 0.001, **P < 0.01, *P < 0.05.

To investigate whether circCDYL exerts anti-oncogenic effects by regulating TJP1 expression level, we co-transfected SK-NEP-1 and G401 cells with circCDYL adenovirus and miR-145-5p mimics. In the co-transfected cells, increased TJP1 proteins induced by overexpressing circCDYL were partially reversed (Figure 6A). Moreover, co-transfection of circCDYL and miR-145-5p partially reversed the effect of circCDYL on proliferation (Figures 6B–E) and migration and invasion (Figures 6F,G). Overall, these data showed that circCDYL plays a regulatory role through the circCDYL/miR-145-5p/TJP1 axis.

## Discussion

WT is a common urinary system carcinoma, and its worldwide morbidity and mortality remain considerable. In this research, we firstly reported that the circular RNA circCDYL was downregulated in WT and that the overexpression of circCDYL suppresses proliferation, migration, and invasion in WT cells.

In recent years, accumulating studies have substantiated that abnormal expression of circRNAs is connected with the generation and progression of various diseases, and can be used as therapeutic targets and biomarkers (Lei et al., 2019). It was previously revealed that circCDYL was upregulated and promoted tumorigenesis in hepatocellular carcinoma (Wei et al., 2020), breast cancer (Liang et al., 2020), multiple myeloma (Chen F. et al., 2020), and mantle cell lymphoma (Mei et al., 2019). In contrast, circCDYL was downregulated in colon cancer (Cui et al., 2019; Wang et al., 2020) and bladder cancer (Sun et al., 2019). Our functional assays revealed that overexpression of circCDYL suppresses proliferation, migration, and invasion in WT cells *in vitro* and *in vivo*, suggesting that circCDYL exerts an anti-oncogenic effect in WT. CircRNAs are identified to be tissue-specific or cell type-specific (Xia et al., 2017), which may explain the expression quantity differences of circCDYL in WT and other tumors.

Previous studies demonstrated that circRNAs can exert as a ceRNA of binding to the specific miRNAs, due to their specific miRNA response elements, and to ulteriorly regulate the target genes (Xiong et al., 2018). MiRNA is a type of single-stranded non-coding RNA and is about 22 nucleotides in length. Accumulating studies have validated that miRNAs play important roles in tumors by directly binding to the 3′UTRs of mRNA and causing their post-transcriptional degradation (Pedroza-Torres et al., 2019). In this study, we demonstrated that circCDYL could sponge miR-145-5p.

miR-145-5p was reported to have antioncogenic properties in several tumors. Previously, the levels of miR-145-5p were reported to be lower in colorectal cancer and decreased miR-145-5p expression promoted the migration, invasion, and epithelial–mesenchymal transition (Chen Q. et al., 2020). Additionally, elevated microRNA-145-5p suppressed the proliferation and migration and facilitated apoptosis of hepatocellular carcinoma cells *in vitro* (Wang et al., 2019). It was demonstrated that ectopic expression of miR-145-5p led to sensitization of breast cancer cells to cisplatin therapy (García-García et al., 2019). Our research proved that miR-145-5p could reduce the expression level of TJP1 in WT. TJP1 was identified as a member of the membrane-associated guanylate kinase homolog family. It maintains epithelial tight junction integrity by interacting with transmembrane proteins and connecting tight junction elements to the cortical actin cytoskeleton (Anderson, 1996; Gottardi et al., 1996). It was reported that TJP1 could promote gastric cancer cell proliferation and motility (Xia et al., 2020). Targeting TJP1 could attenuate cell–cell aggregation and enhance chemosensitivity to doxorubicin in leiomyosarcoma (Lee et al., 2020). In our study, TJP1 was found to exsert anti-oncogenic role in WT.

In conclusion, our research demonstrated that circCDYL can sponge miR-145-5p to upregulate TJP1 expression level, enabling it to restrain cell proliferation, migration, and invasion. These findings indicate that circCDYL/miR-145-5p/TJP1 may be a novel therapeutic target for WT.

## Data Availability Statement

The raw data supporting the conclusions of this article will be made available by the authors, without undue reservation.

## Ethics Statement

The studies involving human participants were reviewed and approved by Guangzhou Women and Children’s Medical Center Ethics Committee. Written informed consent to participate in this study was provided by the participants’ legal guardian/next of kin. The animal study was reviewed and approved by the Animal Management Committee of Guangzhou Medical University.

## Author Contributions

GL and WF designed the study. RZ, WJ, and XG collected the clinical information, performed the experiments, and wrote the manuscript. FD, KF, TZ, and ZL helped in conceiving and/or analyzing the experiments and provided reagents. All authors read and approved the manuscript and agreed to be accountable for all aspects of the research in ensuring that the accuracy or integrity of any part of the work were appropriately investigated and resolved.

## Conflict of Interest

The authors declare that the research was conducted in the absence of any commercial or financial relationships that could be construed as a potential conflict of interest.

## Publisher’s Note

All claims expressed in this article are solely those of the authors and do not necessarily represent those of their affiliated organizations, or those of the publisher, the editors and the reviewers. Any product that may be evaluated in this article, or claim that may be made by its manufacturer, is not guaranteed or endorsed by the publisher.

## Footnotes

1
http://cbio.mskcc.org/microrna_data/miRanda-aug2010.tar.gz

2
http://tiger.dbs.nus.edu.sg/microtar/

3
https://bibiserv.cebitec.uni-bielefeld.de/rnahybrid

## Supplementary Material

Supplementary Figure 1 **(A)** No significant difference of the expression level of CDYL mRNA was detected in WT tumor tissues and their adjacent tissues. **(B)** CircCDYL overexpression did not alter the expression level of CDYL mRNA in SK-NEP-1 and G401 cells. Data represent the mean ± SD from three independent experiments. Student’s *t*-test with two biologically dependent or independent replicates was used to determine statistical significance; ****P* < 0.001, ***P* < 0.01, **P* < 0.05.

Supplementary Figure 2 circCDYL overexpression could lead to S phase reduction **(A)** in SK-NEP-1 **(B)** and G401 **(C)** cells. Data represent the mean ± SD from three independent experiments. Student’s *t*-test with two biologically dependent or independent replicates was used to determine statistical significance; ****P* < 0.001, ***P* < 0.01, **P* < 0.05.

## Abbreviations

circRNA: circular RNA
miRNA: microRNA
RIP: RNA immunoprecipitation
TJP1: tight junction protein 1
FISH: fluorescence *in situ* hybridization
WT: Wilms’ tumor.
