# Supplementary figures and images for: CircCDYL Acts as a Tumor Suppressor in Wilms’ Tumor by Targeting miR-145-5p
Source: Front Cell Dev Biol. 2021 Aug 17;9:668947. doi: 10.3389/fcell.2021.668947 (PMC8415843; doi:10.3389/fcell.2021.668947)

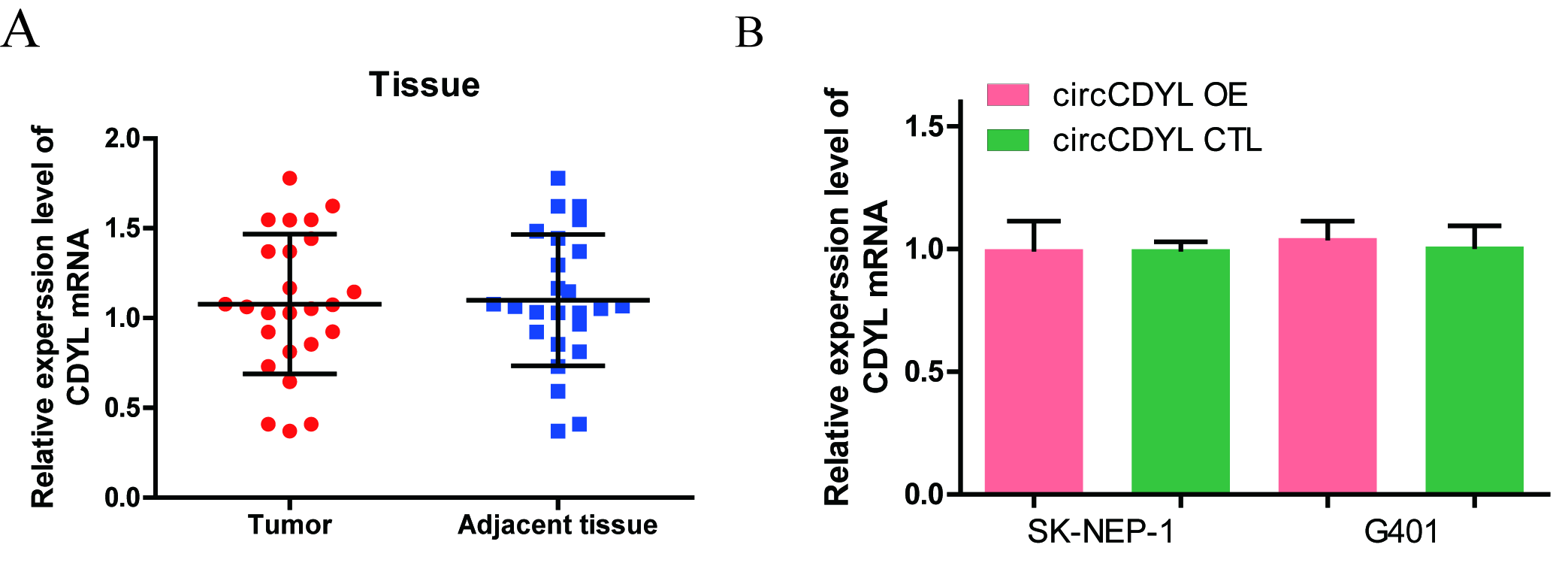

Supplement: Supplementary Figure 1 — (A) No significant difference of the expression level of CDYL mRNA was detected in WT tumor tissues and their adjacent tissues. (B) CircCDYL overexpression did not alter the expression level of CDYL mRNA in SK-NEP-1 and G401 cells. Data represent the mean ± SD from three independent experiments. Student’s t-test with two biologically dependent or independent replicates was used to determine statistical significance; ***P < 0.001, **P < 0.01, *P < 0.05. [file Image_1.TIF]

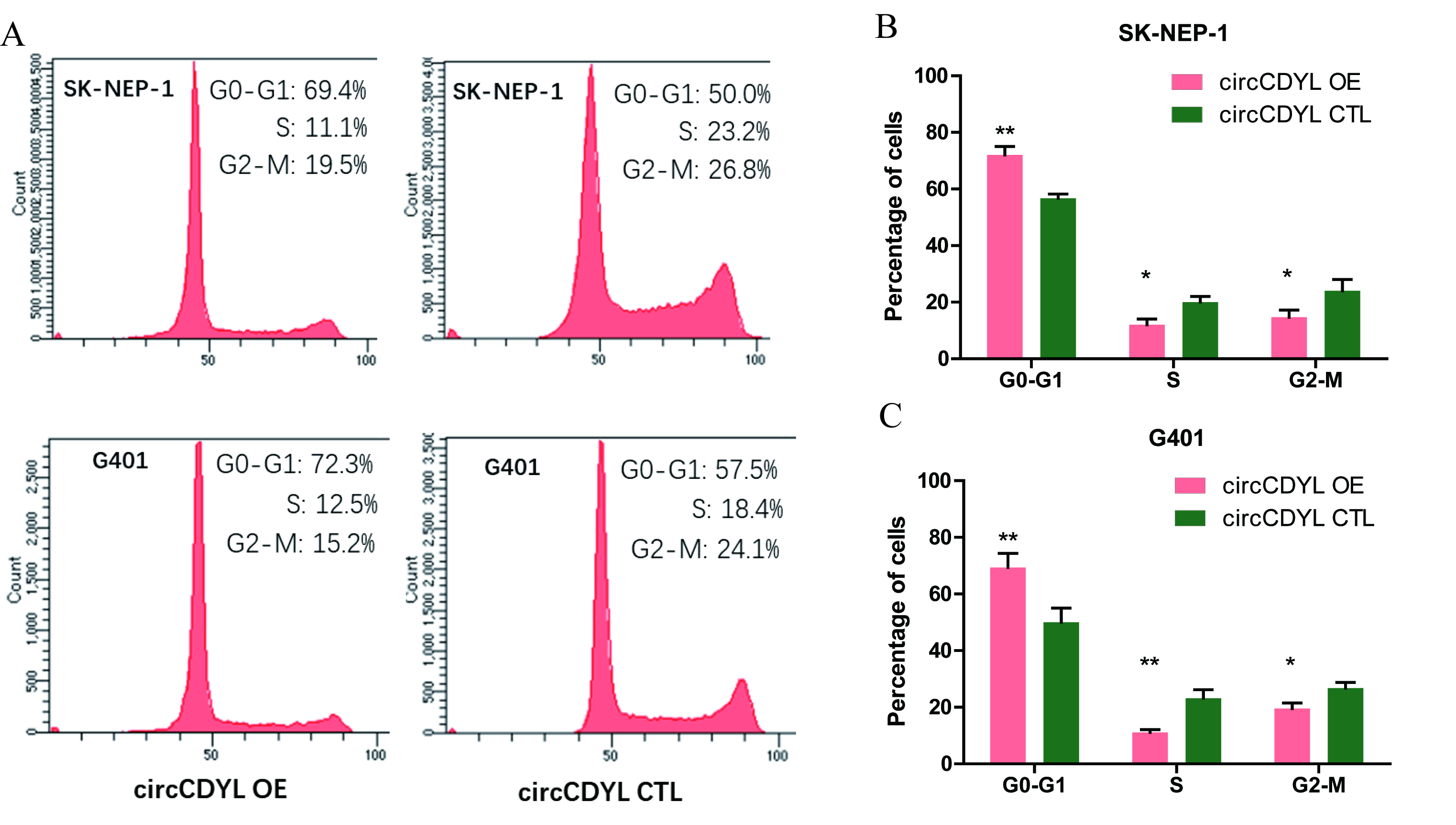

Supplement: Supplementary Figure 2 — circCDYL overexpression could lead to S phase reduction (A) in SK-NEP-1 (B) and G401 (C) cells. Data represent the mean ± SD from three independent experiments. Student’s t-test with two biologically dependent or independent replicates was used to determine statistical significance; ***P < 0.001, **P < 0.01, *P < 0.05. [file Image_2.TIF]
