# Supplementary material for: CircCDYL Acts as a Tumor Suppressor in Wilms’ Tumor by Targeting miR-145-5p
Source: Front Cell Dev Biol. 2021 Aug 17;9:668947. doi: 10.3389/fcell.2021.668947 (PMC8415843; doi:10.3389/fcell.2021.668947)
Supplement: Supplementary file 3 [file Table_1.DOCX]

| β-actin | F | 5’- CCCGAGCCGTGTTTCC-3’ |
| --- | --- | --- |
|  | R | 5’- GTCCCAGTTGGTGACGATGC-3’ |
| circCDYL | F | 5’-AGCCGGTCGGAGCTTTATTG-3’ |
|  | R | 5’-TCCTTTCAACCTTTCCCGTTAAC-3’ |
| TJP1 | F | 5’-AGCCATTCCCGAAGGAGTTG-3’ |
|  | R | 5’-ATCACAGTGTGGTAAGCGCA-3’ |
| U6 | F | 5’-GCTTCGGCAGCACATATACTAAAAT-3’ |
|  | R | 5’-CGCTTCACGAATTTGCGTGTCAT-3’ |
| miR-145-5p | F | 5’- CTCACGGTCCAGTTTTCCCA-3’ |
|  | R | 5’- ACCTCAAGAACAGTATTTCCAGG-3’ |
| U1 | F | 5’-ACTTACCTGGCAGGGGAGATACC-3’ |
|  | R | 5’-CCACTACCACAAATTATGCAGTCG-3’ |
